# Everybody's Page

**Published:** 1888-09-08

**Authors:** 


### Everybody's Page.

Ordinary musical tones range between forty and four thousand vibrations a second. Skilled musicians, it is said, are able to perceive a difference between two notes of as little as 1-64th of a semitone.

A case was reported in the *Medical Times* of 1869 of a copious eruption on the chest arising from wearing a red "chest protector," and in another case a similar effect was produced by aniline dye in a flannel vest.

The use of tobacco is commenced very early in some nations. In "The Voyage of the *Vega*," Captain Nordenskiold states that the Chuckches of all ages are great smokers. He often saw little boys of three years old take a whiff or two of their father's pipe. The women do not smoke, but confine themselves to chewing tobacco, and this they begin very early, in fact, often before they can walk.

Experience has shown that in not a few cases the soluble salts in the water used for personal ablution are a fertile source of irritation-injury to sensitive skins. In some instances the mere substitution of boiled water effects a sensible reduction in the roughness, or other discomfort; but it often occurs that only distilled water will give complete relief, so inimical are the ingredients of hard water to delicate complexions.

The proportion of short-sighted students in Breslau was found by Cohn to be 60 per cent., and amongst the medical students 56 per cent. in 1867, and 57 per cent. in 1881; and he noticed that in the first year of study the proportion is 52 per cent., whilst in the last year it has risen to 64 per cent. He goes so far as to suggest that a term for this special form of myopia should be instituted, and that it should be termed the myopia of examination.

Although the majority of persons are capable of hearing about nine octaves of notes, there are some whose powers are restricted to far less. Wollaston discovered that the capacity for hearing of a friend of his ceased at a note four octaves above the middle E of the pianoforte, although his perception of musical pitch was as correct as that of ordinary ears. The chirping of the cricket, the squeak of the bat, and the chirping of the common house-sparrow are of too high a pitch to be heard by some.

Actors and actresses are no longer subjected to the baneful influences of lead and paint as in days gone by. Wig paste, as it is termed, by reason of its having been originally used exclusively to hide the line the wig made on the forehead, is now employed to coat the entire face, and so preserve it from direct contact with either paint or powder. The wig paste is itself tinted to the various shades required, but it can be further coloured according to taste. It contains nothing more injurious than a little oxide of zinc in combination with some kind of grease, usually tallow. By the application of this paste an actor can model his nose to any shape required.

Some of us have laughed at the schoolboy who, when asked to compose a theme on pins, wrote, "Pins have saved a great many lives by not swallowing them." Perhaps the Pittsville workman who a little while ago fell from the roof of a house and was saved from being dashed to pieces by a pair of electric lighting wires would take the story more seriously. It is said that the two leads were carrying a current of 1,000 volts, alternating currents, which effectually prevented him leaving go until some one came with a long ladder and helped him down. Thus it would appear that the full force of a powerful current, so far from killing him, actually saved the man's life; but he is probably the first person who has received such a current and been thankful for it.

Gregory of Tours affirms that in the royal family of France it was long the peculiar privilege of kings to wear the hair uncut and artfully dressed and curled, everybody else being required to be polled, or cut close round the head, in token of inferiority. To cut off the hair of a son of France under the first race of kings was to declare him excluded from the right of succession, and reduced to the condition of a subject.

As regards brushing the hair, there is a tradition that "you cannot brush the scalp too much nor the hair too little." To say the least of it, this is very questionable. Some scalps will tolerate but little brushing, and even then the brush must be a soft one. Bristle brushes are preferable to wire ones, but constant scratching of the head by any kind of brush or comb is mischievous.

The Venetian ladies of the middle ages are reported to have bleached their hair by wearing it loosely spread out upon the rims of crownless hats, the effect of which exposure to the sun and air was very similar to that now obtained by the application of peroxide of hydrogen, a fairy whose aid is at present invoked, under various familiar aliases, to transform a dark head of hair into a lighter one.

What food the prehoristic people of the Stone Age in Europe ate in their day, has been ascertained in a novel manner. An Englishman took the teeth of a human being of that age, which has been found in recent years, and examined what he found in the dental tartar. After using dilute hydrochloric acid, he examined the sediment, and found portions of the husks of corn, spiral vessels from vegetables, husks of starch, the point of a fish's tooth, a conglomeration of oval cells, probably of ruit, barblets of feathers, epithelial scales, fragments of artilage, and other organic remains.

People who are fond of reading in bed should take warning by the fate of James Parkes, aged 19 years, lately residing at No. 46, Webber Street, Blackfriars. He was very fond of reading in bed, and on Sunday night he retired to bed, taking with him a novel. Whilst reading by the aid of a lamp he dozed off to sleep. Shortly after an alarm of fire was raised, and Parkes was discovered lying in bed in flames, with an upset spirit lamp by his side. The unfortunate youth was terribly burnt on his back and other parts of his body, and subsequently expired in the Leopold Ward at St. Thomas's Hospital.

A good deal of discussion having taken place on the subject of the spread of infectious diseases by means of the books in circulating libraries, the Dresden municipal authorities have had a thorough experimental investigation of this question conducted. A number of much-used volumes from the town library were taken for the purpose. The dust from the leaves and covers was sown in nutrient media and cultures reared, the result being that no microbes belonging to infectious diseases were found—the dust being, in fact, nothing but ordinary dust of a harmless character. Again, the dirtiest leaves in the books were rubbed first with the dry finger and then with the wet finger. In the first place scarcely any microbes were found on the finger; in the second case plenty were found; but all appeared to be of a non-infectious character. Especially is it noted that there were no tubercle bacilli. Lastly, books were soaked for two days in spirit containing 10 per cent. of carbolic acid. This treatment destroyed all the bacilli, and proved harmless to the volumes. The conclusion arrived at was that the danger of circulating libraries spreading infection is very slight, but a recommendation is given to dust books well before reading them, and never to wet the finger in the mouth for the purpose of turning over the leaves.

